# Effects of omega-3 polyunsaturated fatty acid supplementation on cognitive functioning in youth at ultra-high risk for psychosis: secondary analysis of the NEURAPRO randomised controlled trial

**DOI:** 10.1192/bjo.2022.572

**Published:** 2022-09-08

**Authors:** Nicholas Cheng, Alison McLaverty, Barnaby Nelson, Connie Markulev, Miriam R. Schäfer, Maximus Berger, Nilufar Mossaheb, Monika Schlögelhofer, Stefan Smesny, Ian B. Hickie, Gregor E. Berger, Eric Y. H. Chen, Lieuwe de Haan, Dorien H. Nieman, Merete Nordentoft, Anita Riecher-Rössler, Swapna Verma, Rebekah Street, Andrew Thompson, Hok Pan Yuen, Robert Hester, Alison Ruth Yung, Patrick D. McGorry, Kelly Allott, G. Paul Amminger

**Affiliations:** Orygen, Centre for Youth Mental Health, The University of Melbourne, Australia; Melbourne School of Psychological Sciences, The University of Melbourne, Australia; Orygen, Centre for Youth Mental Health, The University of Melbourne, Australia; and Department of Psychiatry and Psychotherapy, University of Bern, Switzerland; Department of Psychiatry and Psychotherapy, Medical University of Vienna, Austria; BioPsyC Biopsychosocial Corporation, Vienna, Austria; Department of Psychiatry and Psychotherapy, Jena University Hospital, Germany; Brain and Mind Centre, The University of Sydney, Australia; Child and Adolescent Psychiatric Service, Canton of Zurich, Switzerland; Department of Psychiatry, University of Hong Kong, Hong Kong; Department of Psychiatry, Academic Medical Center, The Netherlands; Mental Health Centre Copenhagen, Department of Clinical Medicine, Copenhagen University Hospital, Denmark; Faculty of Medicine, University of Basel, Switzerland; Early Psychosis Intervention, Institute of Mental Health, Singapore; Institute for Mental and Physical Health and Clinical Translation (IMPACT), Deakin University, Australia; and School of Health Sciences, University of Manchester, UK; Orygen, Centre for Youth Mental Health, The University of Melbourne, Australia; and Melbourne School of Psychological Sciences, The University of Melbourne, Australia

**Keywords:** Cognition, clinical high risk, early intervention, randomised controlled trial, psychotic disorders

## Abstract

**Background:**

Cognitive impairments are well-established features of psychotic disorders and are present when individuals are at ultra-high risk for psychosis. However, few interventions target cognitive functioning in this population.

**Aims:**

To investigate whether omega-3 polyunsaturated fatty acid (*n*−3 PUFA) supplementation improves cognitive functioning among individuals at ultra-high risk for psychosis.

**Method:**

Data (*N* = 225) from an international, multi-site, randomised controlled trial (NEURAPRO) were analysed. Participants were given omega-3 supplementation (eicosapentaenoic acid and docosahexaenoic acid) or placebo over 6 months. Cognitive functioning was assessed with the Brief Assessment of Cognition in Schizophrenia (BACS). Mixed two-way analyses of variance were computed to compare the change in cognitive performance between omega-3 supplementation and placebo over 6 months. An additional biomarker analysis explored whether change in erythrocyte *n*−3 PUFA levels predicted change in cognitive performance.

**Results:**

The placebo group showed a modest greater improvement over time than the omega-3 supplementation group for motor speed (*η_p_*^2^ = 0.09) and BACS composite score (*η_p_*^2^ = 0.21). After repeating the analyses without individuals who transitioned, motor speed was no longer significant (*η_p_*^2^ = 0.02), but the composite score remained significant (*η_p_*^2^ = 0.02). Change in erythrocyte n-3 PUFA levels did not predict change in cognitive performance over 6 months.

**Conclusions:**

We found no evidence to support the use of omega-3 supplementation to improve cognitive functioning in ultra-high risk individuals. The biomarker analysis suggests that this finding is unlikely to be attributed to poor adherence or consumption of non-trial *n*−3 PUFAs.

Cognitive impairments are well-established features of psychotic disorders and are present when individuals are at ultra-high risk (UHR) for developing a full-threshold disorder.^1^ Cognitive deficits have shown to be a pre-existing risk factor for transition to psychosis among UHR individuals, particularly moderate impairments in verbal fluency, working memory, processing speed and verbal memory.^1,2^ Despite this, few interventions target cognitive functioning in UHR populations.^3^ Omega-3 polyunsaturated fatty acids (*n*−3 PUFAs), such as eicosapentaenoic acid (EPA) and docosahexaenoic acid (DHA), are primarily consumed through diet and have anti-inflammatory and antioxidant properties.^4^ *n*−3 PUFA supplementation has thus been considered as a potential intervention for improving UHR outcomes,^5^ given the lower concentrations of EPA^6^ and higher inflammation and oxidative stress in this population relative to healthy controls.^7,8^ *n*−3 PUFA supplementation has previously shown a long-term reduction in the transition rate to psychosis and improvements in functioning in UHR individuals,^9^ although these findings were not replicated in a recent double-blind, randomised controlled trial (North America, Europe, Australia Prodrome Study (NEURAPRO)).^5,10^

## Potential benefits of *n*−3 PUFA supplementation on cognitive functioning

*n*−3 PUFA supplementation has shown to improve attention, processing speed and working memory in ageing populations,^11^ individuals with mild cognitive impairment^12^ and young people with attention-deficit hyperactivity disorder.^4^ Although evidence in UHR individuals is minimal, a small positive association between verbal fluency, EPA and its precursor alpha-linolenic acid, and between working memory and the omega-3/omega-6 ratio, has been observed.^13^ One randomised controlled trial of 162 individuals with schizophrenia and schizoaffective disorder found no impact of *n*−3 PUFA supplementation (500 mg/day EPA over 12–16 weeks) on cognitive functioning.^14^ The authors suggested that supplementation may be less effective in their study sample, with most participants being middle-aged (mean age of 40 years) and unwell for two decades. Thus, *n*−3 PUFA supplementation may be more effective in individuals with less severe illness or younger people who are earlier in the illness course. One meta-analysis found a beneficial effect of EPA-rich *n*-3 PUFA supplementation on working memory, shifting and flexibility, and problem-solving in young people aged up to 25 years, particularly those with pre-existing cognitive impairments or a clinical condition.^15^ Thus, the benefits of *n*−3 PUFA supplementation on cognitive functioning may be stronger in UHR individuals, given that this population typically presents with milder cognitive impairments,^16^ is younger^17^ and is less severely ill compared with those with established psychotic disorders.

## Aim

The present study is a secondary analysis of the NEURAPRO trial.^5^ We aimed to investigate the impact of *n*−3 PUFA supplementation on cognitive functioning in UHR individuals. It was hypothesised that UHR individuals who receive *n*−3 PUFA supplementation would demonstrate superior cognitive functioning after 6 months, compared with individuals who received a placebo.

## Method

### Setting and design

The complete NEURAPRO trial (Australian and New Zealand Clinical Trial Registry identifier 12608000475347) methodology can be found elsewhere.^5^ Participants were aged 13–40 years and, in addition to sustained low or reduction in psychosocial functioning, met at least one of three UHR categories according to the Comprehensive Assessment of At-Risk Mental State: (a) attenuated psychotic symptoms, (b) brief limited intermittent psychotic symptoms and/or (c) vulnerability group (i.e. schizotypal personality disorder or a first-degree relative with a psychotic disorder).^18^ Participants without 6-month cognition data were excluded from the present analysis. Over 6 months, the intervention group received *n*−3 PUFA supplementation and the control group received a placebo. Both groups received adjunctive cognitive–behavioural case management (CBCM). Participants were recruited from clinics in Melbourne, Sydney, Vienna, Singapore, Basel, Hong Kong, Copenhagen, Zurich, Jenna and Amsterdam.

The authors assert that all procedures contributing to this work comply with the ethical standards of the relevant national and institutional committees on human experimentation and with the Helsinki Declaration of 1975, as revised in 2008. All procedures involving human participants were approved by the Melbourne Health Human Research Ethics Committee (approval number 2008.628) and the local ethics committees relevant to each site. All participants (parent/guardian for participants under 18 years of age) provided written informed consent to participate in this study.

### Randomisation procedure

Participants were randomised to *n*−3 PUFA supplementation or placebo via an online data management system. Randomisation was stratified according to site and levels of depressive symptoms according to the Montgomery–Åsberg Depression Rating Scale (MADRS),^19^ as depressive symptoms may contribute to illness progression in UHR.^20^

### Interventions

Participants were dispensed bottles of gelatine capsules and asked to take four capsules per day. Participants in the *n*-3 PUFA group were given capsules containing 0.65–0.75 g of concentrated marine fish oil totalling approximately 1.4 g of *n*-3 PUFA or 840 mg of EPA and 560 mg of DHA. The placebo capsules resembled the fish oil capsules in appearance and contained 1% fish oil to mimic taste. They also contained 0.65–0.75 g of paraffin oil as this does not interfere with PUFA metabolism. Adherence was assessed by capsule count. Participants were considered adherent if <25% of capsules were returned. Participants in both groups received up to 20 sessions of CBCM as a co-intervention. In addition, participants could receive selective serotonin reuptake inhibitors to treat depression and/or benzodiazepines for anxiety or insomnia based on clinical discretion in their standard care. Benzodiazepine use was kept to a minimum for short periods *pro re nata*, and did not differ between the *n*−3 PUFA and the placebo group. Antipsychotic or mood-stabilising medications were not permitted in the trial.

### Measures

#### Participant characteristics

Demographic information collected at baseline included age, gender, ethnicity, highest level of education, type of work, subjective health (six-point scale from excellent to very poor) and body mass index. IQ was measured with the Wechsler Adult Intelligence Scale Third Edition Short Form.^21^ Smoking status was determined by the World Health Organization Alcohol, Smoking and Substance Involvement Screening Test.^22^ Levels of positive, negative and depressive symptoms were measured with the Brief Psychiatric Rating Scale psychosis subscale,^23^ Scale for the Assessment of Negative Symptoms and the MADRS. The Social and Occupational Functioning Assessment Scale was used to measure general psychosocial functioning.^24^ All instruments were translated to local languages (English, Danish, Dutch, German and Chinese) by NAATI-accredited translators.^5^

#### Cognitive assessment

The Brief Assessment of Cognition in Schizophrenia (BACS)^25^ composite score was used to measure global cognitive function. The BACS was also used to measure six cognitive domains: verbal memory (verbal memory task), working memory (digit sequencing task), motor speed (token motor task), verbal fluency (semantic fluency and letter fluency tasks), processing speed (symbol coding task) and executive function (Tower of London test). The BACS was administered by research assistants who were blinded from the participant's treatment allocation. To ensure consistency in administration across sites, all research assistants were trained to administer the BACS according to a standardised procedure.^26^ All tasks were administered on pen and paper, and participants were attentive when assessed. The BACS raw scores were standardised for age and gender and reported as *z*-scores.^25^

#### Erythrocyte *n*−3 PUFA measures

Fasting blood samples were collected from participants at baseline and 6 months (end of intervention period). Samples underwent preliminary processing, including extraction of erythrocytes, and were stored at −80°C until analysed. Erythrocyte *n*−3 PUFA levels are an accurate biological marker for dietary intake of *n*−3 PUFA and closely reflect the fatty acid content of neuronal membranes.^27,28^ The molecular percentage of total fatty acid levels of EPA, DHA and omega-3 index (EPA + DHA) were measured by gas chromatography. The phosphatidylethanolamine fraction, located on the inner side of the cell membrane, was used to determine the omega-3 fatty acid content, because of their high abundancy in the lipid raft.^29^ All data were screened for implausible values that may indicate problems with the sample, such as degradation.

### Statistical analysis

Analyses were completed with SPSS (version 23 for Windows). A mixed two-way analysis of variance (ANOVA) was conducted, including only those with both baseline and follow-up cognition data. Participants with and without cognition data were compared on baseline variables to determine whether there were systematic differences between them.

Homogeneity of variance was assessed with Levene's test, and difference scores between baseline and follow-up scores on cognitive function were inspected for normality. Scores for each cognitive domain at baseline and 6-month follow-up were included as the within-participant factors, and the intervention group was included as the between-participants factor. The effect size was calculated with partial eta squared (*η_p_*^2^). The analyses were repeated by excluding participants who transitioned to psychosis over the 6-month period, to ensure transition was not a confounding variable. Significant results were further analysed by including treatment adherence as a covariate.

## Results

### Participant characteristics

**Fig. 1:**
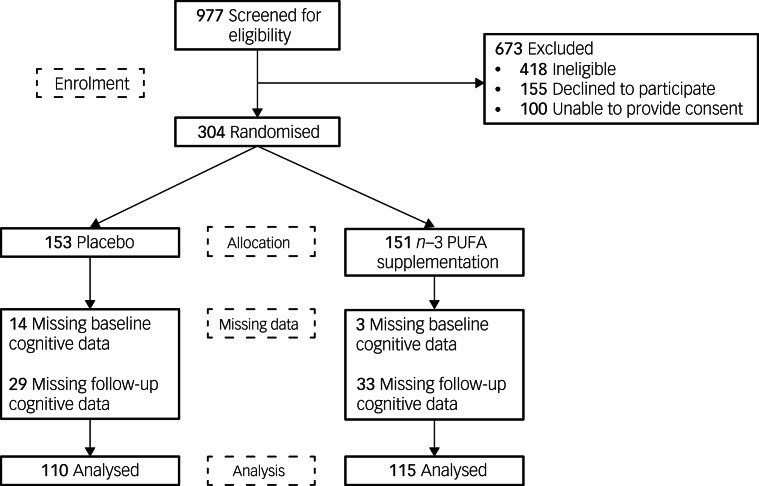
Flow of participants. *n*−3 PUFA, omega-3 polyunsaturated fatty acid.

Figure 1 displays the flow of participants (recruited between March 2010 and September 2013), with 225 included in the current analysis. The analysed groups were compared with the original randomised groups on key demographic variables. The participants who had both baseline and follow-up cognition data were more likely to be non-smokers (*χ*² = 4.11, *P* = 0.043, odds ratio 1.84).

Table 1 shows the demographic and clinical characteristics for both the placebo and *n*−3 PUFA groups. The groups did not significantly differ on these variables at baseline. Regarding cognitive performance, there were no significant differences in mean composite score and the six domains at baseline (see Table 2).

**Table 1:** Baseline participant characteristics in the omega-3 polyunsaturated fatty acid supplementation and placebo group

	*n*−3 PUFA (*n* = 110)	Placebo (*n* = 115)	
	(%)	(%)	*P*-value
Female	49.3	61.2	0.154
Ethnicity			
Caucasian	81.11	84.1	
African	2.03	1.4	
Asian	13.51	11.6	
Other	3.37	2.9	0.798
Smoking	60.1	51.7	0.105
	Mean (s.d.)	Mean (s.d.)	
Age (years)	19.08 (4.72)	18.79 (4.09)	0.627
IQ estimate	103.81 (15.65)	102.79 (14.04)	0.611
Education/training (years)	10.41 (3.29)	10.45 (3.20)	0.917
BMI	24.45 (6.07)	23.70 (4.94)	0.340
Subjective health	3.71 (1.11)	3.89 (.94)	0.204
BPRS total	41.32 (9.98)	40.84 (9.07)	0.703
SANS total	18.77 (13.28)	17.55 (12.47)	0.481
MADRS	19.50 (8.52)	19.69 (9.19)	0.875
SOFAS	53.59 (11.51)	54.09 (12.57)	0.756

*n*−3 PUFA, omega-3 polyunsaturated fatty acid; BMI, body mass index; BPRS, Brief Psychiatric Rating Scale; SANS, Scale for the Assessment of Negative Symptoms; MADRS, Montgomery–Åsberg Depression Rating Scale; SOFAS, Social and Occupational Functioning Assessment Scale.

**Table 2:** Results of the mixed two-way analysis of variance showing the main effects for group and time, and the time×group interaction

	Mean (s.d.)					Group			Time			Time×group		
	Omega-3		Placebo		*d.f.*	*F-*value	*P-*value	*η*_p_^2^	*F-*value	*P-*value	*η*_p_^2^	*F-*value	*P-*value	*η*_p_^2^
	Baseline	Follow-up	Baseline	Follow-up										
Composite score	–0.38 (1.37)	–0.10 (1.40)	–0.54 (1.33)	0.04 (1.25)	1, 222	0.05	0.829	<0.001	58.61	**<0**.001**	0.21	4.62	**0**.033*	0.02
Verbal memory	–0.09 (1.51)	–0.28 (1.39)	–0.26 (1.53)	0.27 (1.24)	1, 223	0.28	0.594	0.001	39.97	**<0**.001**	0.15	1.29	0.240	0.006
Working memory	–0.37 (1.10)	–0.33 (1.16)	–0.40 (1.11)	–0.24 (1.04)	1, 223	0.65	0.799	<0.001	3.30	0.071	0.02	1.30	0.256	0.006
Motor speed	–0.37 (1.17)	–0.18 (1.27)	–0.49 (1.07)	0.018 (1.11)	1, 222	0.09	0.762	<0.001	23.11	**<0**.001**	0.09	4.69	0.031*	0.02
Verbal fluency	–0.53 (1.12)	–0.42 (1.31)	–0.48 (1.26)	–0.30 (1.21)	1, 223	0.30	0.585	0.001	8.06	**0**.005*	0.12	0.38	0.540	0.002
Processing speed	–0.23 (1.14)	0.07 (1.34)	–0.21 (1.27)	0.23 (1.26)	1, 223	0.34	0.559	0.002	42.73	**<0**.001**	0.16	1.74	0.189	0.008
Executive functioning	0.22 (1.24)	0.27 (0.81)	0.19 (1.03)	0.29 (0.93)	1, 223	0.001	0.981	<0.001	1.38	0.242	0.006	0.11	0.745	<0.001

Mean and s.d. are reported in *z*-scores standardised for age and gender. *η_p_*^2^, partial eta squared. Bolded *P*-values indicate statistical significance.

*P < 0.05.

**P < 0.001.

### Primary analysis

The results of the mixed two-way ANOVAs are displayed in Table 2. There was no evidence of violating the homogeneity of variance, and differences between baseline and 6-month follow-up cognitive scores appeared normally distributed. A statistically significant increase in cognitive function from baseline to 6-month follow-up was observed in verbal memory, motor speed, verbal fluency, processing speed and the composite score. The time×group interaction was significant for motor speed and the composite score, with the placebo group showing a significantly greater improvement over time than the *n*−3 PUFA group.

Of the participants included in the analysis, six transitioned to psychosis over the 6-month intervention period (*n =* 3 from the placebo group, *n* = 3 from the *n*−3 PUFA group). The analysis was repeated by excluding these participants to ensure transition was not a confounding variable. The effect size of the time×group interaction for motor speed decreased and was no longer statistically significant (*F*(1, 216) = 3.84, *P* = 0.051, *η_p_*^2^ = 0.02). There was also a reduction in effect size for the composite score; however, this remained statistically significant (*F*(1, 216) = 4.03, *P* = 0.046, *η_p_*^2^ = 0.02). Notably, the *P*-values for both analyses are close to the alpha value of 0.05. Interpretation of the statistical significance of the main and interaction effects were unchanged for all other cognitive domains.

Adherence data was available for all participants included in the mixed two-way ANOVA. The adherence for the placebo group (52.7%) and the adherence for supplementation (51.3%) did not significantly differ (*χ*^2^(1, *N* = 225) = 0.05, *P* = 0.831). Excluding individuals who transitioned, analysis of the composite score was repeated including adherence as a covariate. The time×group interaction remained marginally significant with the inclusion of the covariate (*F*(1, 215) = 4.02, *P* = 0.046, *η_p_*^2^ = 0.02). Both the main effect of adherence (*F*(1, 215) = 1.47, *P* = 0.227, *η_p_*^2^ = 0.007) and its interaction with time (*F*(1, 215) = 0.01, *P* = 0.908, *η_p_*^2^ < 0.001) were not significant.

### Biomarker analysis

Given the non-significant effect of *n*−3 PUFA supplementation on cognitive performance, and the small effect sizes for motor speed and the composite score, we additionally examined whether change in erythrocyte *n*−3 PUFA levels, regardless of group allocation in the NEURAPRO trial, could predict change in cognitive performance. This was critical, given the difficulty in determining the source of *n*−3 PUFA consumption and the potential for participants in the placebo group to consume non-study *n*−3 PUFAs (e.g. dietary intake or non-trial supplements). Linear regression models were computed by regressing each BACS cognitive measure onto change in the omega-3 index (molecular percentage of total fatty acid levels of EPA + DHA) between baseline and 6-month follow-up. The seven models were adjusted for baseline omega-3 index and the respective baseline cognitive score.

The mean omega-3 index was 12.17 (s.d. 1.94) at baseline (mean 12.09, s.d. 1.71 for the supplementation group; mean 12.26, s.d. 2.16 for the placebo group) and 14.08 (s.d. 4.20) at the 6-month follow-up (mean 15.91, s.d. 4.31 for the supplementation group; mean 12.10, s.d. 3.04 for the placebo group). Change in the omega-3 index between baseline and the 6-month follow-up did not significantly predict change in any of the cognitive outcome measures over the follow-up period.

## Discussion

To the best of our knowledge, this is the largest study investigating the role of *n*−3 PUFAs on cognitive functioning in UHR individuals. We did not find evidence to suggest that *n*−3 PUFA supplementation benefits cognitive functioning on any domain in the present sample. An unexpected and slightly larger improvement in motor speed and the composite score was observed in the placebo compared with the *n*−3 PUFA group, although this finding did not remain significant for motor speed after including adherence as a covariate. Additionally, our biomarker analysis measuring erythrocyte *n*−3 PUFA levels did not predict change in any cognitive domain. Thus, we did not find any evidence that *n*−3 PUFAs benefited cognitive function in this UHR sample.

The benefit observed in the placebo over *n*−3 PUFA supplementation in the composite score and motor speed was unexpected and does not reflect findings from previous literature.^30,31^ However, when excluding transitioned cases, the finding for motor speed was no longer significant, suggesting that this effect may have been substantially driven by a small number of cases. The result for the composite score, however, was more robust. Indeed, a previous correlation analysis of the present sample found a negative association between DHA and the composite score,^13^ although it is unlikely that *n*−3 PUFA supplementation would inhibit cognitive function via the elevation of this biomarker and a negative correlation was not observed for EPA in that study. Alternatively, we speculated that the result may be attributed to the consumption of non-study *n*−3 PUFAs in the placebo group, and that the low adherence rate could have amplified this effect. This is considered a core limitation in omega-3 trials, given that *n*−3 PUFA supplementation is more accessible than most trial substances and its benefits are well known.^32^ However, given the theoretical basis of previous literature and the small effect size and marginal significance of this result, it is likely that the benefit of the placebo is attributable to chance.

Previous evidence has suggested that *n*−3 PUFA supplementation can benefit cognitive function, although findings have been mixed.^11,31,33,34^ Several studies demonstrate a benefit among young clinical populations and individuals with pre-existing cognitive impairments.^4,11,12,15^ Given that UHR individuals tend to be young^17^ and experience cognitive difficulties,^16^ it is possible that our sample size lacked statistical power to detect differences, particularly given the small effect sizes. Nevertheless, mean performance within all cognitive domains were within one standardised *z*-score and mean IQ estimates were within the average range, indicating that cognitive impairment may be mild. It may be that the benefits of *n*−3 PUFAs on cognitive functioning are more likely in UHR individuals with more severe cognitive impairment than what was present in this sample. Nevertheless, *n*−3 PUFA supplementation has not previously demonstrated a benefit on cognitive functioning among individuals with chronic psychosis,^14^ and there is inconsistent evidence for those with neurodevelopmental disorders,^4,34^ both of which involve populations that typically demonstrate severe cognitive impairment.^35^ Indeed, a previous analysis of this sample did not show associations between *n*−3 PUFAs and most cognitive domains.^13^ There was only one small positive correlation between verbal fluency and EPA and alpha-linolenic acid, although verbal fluency did not appear to benefit from supplementation in the present analysis. Thus, it is also possible that the UHR population is one of several clinical groups that do not appear to benefit from *n*−3 PUFA supplementation.^14,34^

The present sample improved in the composite score, verbal memory, motor speed, verbal fluency and processing speed between baseline and follow-up. Improvements in cognitive functioning are commonly observed in UHR samples, albeit as a stable deficit (i.e. normative growth rate with constant impairment) or lag (i.e. attenuated improvement with increasing impairment over time) compared with healthy populations^16,36^ The improvements in this sample may be partially attributed to treatment (e.g. CBCM and/or antidepressants for some individuals) and maturation of adolescent participants. Antidepressants have previously been found to benefit cognitive function in UHR individuals.^37^ It is possible that treatment and maturation created a ceiling effect that minimised any additional benefit of *n*−3 PUFAs. Further, *n*−3 PUFAs are generally considered to exert a neuroprotective effect,^33^ particularly for long-term clinical outcomes (e.g. 12 months) in individuals with higher blood level *n*−3 PUFAs at baseline.^38^ This has several implications for our findings. First, the benefit of *n*−3 PUFAs may more likely manifest as a protective rather than cognitive-enhancing mechanism against cognitive deterioration. It is possible that the neuroprotective effects of *n*−3 PUFAs are minimal or no longer required, given that cognitive functioning is already improving and cognitive impairment is mild. Second, cognitive deterioration has been observed over longer-term analyses of UHR samples,^36^ and transition can occur up to 10 years after initial contact,^39^ meaning that longer-term supplementation may be required to gain a benefit. Third, there may have been attrition in individuals who are more severely unwell, and who experience greater cognitive impairment or deterioration. Thus, we may not have captured individuals who would have been more receptive to the benefits of neuroprotection. Future studies should track cognitive function over a long-term period to determine if supplementation could provide a long-term neuroprotective benefit, particularly in UHR individuals with greater baseline cognitive impairment or those who experience cognitive deterioration.

### Limitations

The limitations of this study must be acknowledged. First, there were no healthy controls. Consequently, we could not determine the extent of cognitive impairment over time in our sample, including whether the overall improvements represented a lag, static deficit or even catch-up to normative levels. This is critical as we cannot determine whether the results are specific to UHR individuals, although previous studies have rarely demonstrated a benefit of *n*−3 PUFA supplementation on cognitive functioning in healthy populations.^33,34^ It is also unclear whether the level of cognitive functioning in our sample was severe enough to yield a similar benefit from *n*−3 PUFA supplementation as other clinical populations,^4,11,12^ although the age-corrected *z*-score estimates suggest that cognitive impairments may be mild in this sample. It is possible that the inclusion of unimpaired participants diluted the potential benefits of *n*−3 PUFAs.

To minimise the confounds of non-trial *n*−3 PUFAs, we excluded individuals who had taken *n*−3 PUFA supplementation before the study.^32^ However, dietary intake of *n*−3 PUFAs and non-study supplementation was not assessed or controlled during the trial. This would be useful for objectively determining whether the supplementation group maintains higher levels of *n*−3 PUFAs relative to the placebo group during the trial. Nevertheless, our analysis of erythrocyte *n*−3 PUFA levels minimises this limitation as this biomarker has been shown to be stable over time. Thus, erythrocyte *n*−3 PUFA levels examined at the end of the intervention period reliably represent levels over the previous 2–3 months. Furthermore, individuals with lower baseline EPA and DHA levels have shown to be more receptive to medium-term benefits of supplementation,^38^ and those with higher baseline *n*−3 PUFAs also experience long-term protective effects.^38^ There may be a non-linear dose–response relationship between *n*−3 PUFAs and cognitive functioning that could not be investigated in our data-set because it contained only two time points. Future studies may consider targeted *n*−3 PUFA prescriptions for specific baseline *n*−3 PUFA profiles, and examine potential non-linear relationships by examining cognitive functioning over more than two time points.

A strength of the study was the large sample size, garnered from multiple sites internationally, which increases the generalisability of the results. However, this may have also increased the heterogeneity of the UHR sample. For example, there are dietary variations between countries, and erythrocyte *n*−3 PUFA levels are highly dependent on diet.^40^ This may have amplified any confounds based on non-study *n*−3 PUFA consumption.

### Clinical implications

We did not find support for the use of *n*−3 PUFA supplementation as an intervention for enhancing cognitive functioning in individuals who are at UHR for psychosis. It is possible that individuals at UHR for psychosis experience mild cognitive impairment, leading to a bigger risk of a ceiling effect. It is also possible that *n*−3 PUFAs exert minimal benefits alongside other interventions, such as CBCM and antidepressant medication. Practitioners are therefore prompted to seek alternative intervention strategies for cognitive functioning in UHR individuals, and more work is needed to identify an effective treatment. Future research may need to investigate whether *n*−3 PUFA supplementation is a viable intervention for cognitive impairment in a subgroup of UHR individuals who experience cognitive deterioration or poor cognitive functioning.

## Data Availability

The data that support the findings of this study are available from the corresponding author, N.C., upon reasonable request.
